# Massive mitochondrial DNA content in diplonemid and kinetoplastid protists

**DOI:** 10.1002/iub.1894

**Published:** 2018-10-06

**Authors:** Julius Lukeš, Richard Wheeler, Dagmar Jirsová, Vojtěch David, John M. Archibald

**Affiliations:** ^1^ Institute of Parasitology Biology Centre, Czech Academy of Sciences České Budějovice (Budweis) Czech Republic; ^2^ Faculty of Science University of South Bohemia České Budějovice (Budweis) Czech Republic; ^3^ Sir William Dunn School of Pathology University of Oxford Oxford UK; ^4^ Department of Biochemistry and Molecular Biology Dalhousie University Halifax Canada

**Keywords:** DNA content, kinetoplast DNA, mitochondrial DNA, protist

## Abstract

The mitochondrial DNA of diplonemid and kinetoplastid protists is known for its suite of bizarre features, including the presence of concatenated circular molecules, extensive *trans*‐splicing and various forms of RNA editing. Here we report on the existence of another remarkable characteristic: hyper‐inflated DNA content. We estimated the total amount of mitochondrial DNA in four kinetoplastid species (*Trypanosoma brucei*, *Trypanoplasma borreli*, *Cryptobia helicis*, and *Perkinsela* sp.) and the diplonemid *Diplonema papillatum*. Staining with 4′,6‐diamidino‐2‐phenylindole and RedDot1 followed by color deconvolution and quantification revealed massive inflation in the total amount of DNA in their organelles. This was further confirmed by electron microscopy. The most extreme case is the ∼260 Mbp of DNA in the mitochondrion of *Diplonema*, which greatly exceeds that in its nucleus; this is, to our knowledge, the largest amount of DNA described in any organelle. *Perkinsela* sp. has a total mitochondrial DNA content ~6.6× greater than its nuclear genome. This mass of DNA occupies most of the volume of the *Perkinsela* cell, despite the fact that it contains only six protein‐coding genes. Why so much DNA? We propose that these bloated mitochondrial DNAs accumulated by a ratchet‐like process. Despite their excessive nature, the synthesis and maintenance of these mtDNAs must incur a relatively low cost, considering that diplonemids are one of the most ubiquitous and speciose protist groups in the ocean. © 2018 The Authors. IUBMB Life published by Wiley Periodicals, Inc. on behalf of International Union of Biochemistry and Molecular Biology., 70(12):1267–1274, 2018

**Abbreviations**
AT: adenine and thymine
bp: base pairs
CNE: constructive neutral evolution
DAPI: 4′,6‐diamidino‐2‐phenylindole
GC: guanine and cytosine
gRNA: guide RNA
kDNA: kinetoplast DNA
mtDNA: mitochondrial DNA

## INTRODUCTION

Mitochondrial genomes come in all shapes and sizes. Much of our detailed knowledge of the diversity and evolution of this endosymbiotically derived organelle stems from Metazoa, but in fact the boundaries of this diversity are to be found among plants and the unicellular eukaryotes (protists) belonging to the supergroup Excavata 1. The mitochondrial genomes of flowering plants range from ~200 kb to over 10 Mb, have very low gene densities, undergo frequent rearrangements associated with the gain or loss of intergenic regions 2, 3, 4 and are capable of acquiring, via horizontal gene transfer, whole mitochondrial genomes from other organisms 5. The mitochondrial genomes of excavate protists are unusual in other ways. While jakobids harbor the most gene‐rich mitochondrial DNA known 6, it is the mitochondrial genomes of Euglenozoa—a diverse group of aerobic excavates composed of photosynthetic Euglenida, heterotrophic free‐living Diplonemea, and parasitic Kinetoplastea—that exhibit a range of unique features. The mitochondrial DNA (or kinetoplast DNA) of the human parasite *Trypanosoma brucei* is amongst the best‐studied non‐nuclear genomes. Composed of thousands of mutually interlocked minicircles and dozens of maxicircles, this single densely packed kDNA network requires highly complex machinery for its maintenance and expression 7. The transcripts of most maxicircle protein‐coding genes undergo RNA editing in the form of uridine insertions and/or deletions 8, 9. These post‐transcriptional events are specified by hundreds of minicircle‐encoded guide (g) RNAs, which along with numerous large protein complexes produce a mere dozen mitochondrial proteins 10. Recent genomic and transcriptomic evidence confirms earlier predictions 11 that uridine‐indel RNA editing is present in all Kinetoplastea 12, with the notable exception of petite mutants of *T. brucei*, who lack the mitochondrial genome as such 13.

While the kinetoplast DNA of *T. brucei* and all related parasitic trypanosomatids exists as a periflagellar densely packed network, the mitochondrial DNA of parasitic and free‐living bodonids, a sister group of trypanosomatids, exists in various forms 14. Electron microscopy and molecular studies have shown that it is usually dispersed throughout much of the mitochondrial lumen, either uniformly or condensed in multiple foci. Its structure also varies, ranging from free circles and small catenanes to long linear or circular molecules. Invariably, however, the bodonids carry much more DNA in their mitochondria than do the trypanosomatids 12, 15, 16, 17.

The mitochondrial DNAs of the Euglenida and Diplonemea, which are sister groups of kinetoplastids, are strikingly different. While the free‐living alga *Euglena gracilis* contains a small mitochondrial genome with a canonical set of protein‐coding genes whose transcripts are apparently not post‐transcriptionally modified 18, the mitochondrial DNA of the heterotrophic *D. papillatum* is truly bizarre. It is composed of thousands of DNA circles, which possess protein‐coding genes broken into tens of fragments. In order to produce functional mRNAs, the products of these broken genes must be *trans*‐spliced into contiguous sequences (often joined by stretches of post‐transcriptionally added uridines) and heavily edited by nucleotide substitutions 19, 20, 21. In other diplonemid species, the complexity of *trans*‐splicing and RNA editing appears even higher than in *D. papillatum* 22, 23. The kinetoplastids and diplonemids appear to illustrate parallel evolution of extremely complex mitochondrial RNA processing machineries not seen in other eukaryotes. Here we show that these two lineages are also remarkable in the total amount of DNA found in their mitochondria.

## MATERIALS AND METHODS

*Trypanosoma brucei*, *Trypanoplasma borreli*, *Perkinsela* sp., and *D. papillatum* were cultivated as described previously 13, 17, 24, 25, while *Cryptobia helicis* was dissected from the receptacula seminis of the snail *Helix pomatia* as described elsewhere 16.

DNA staining for color deconvolution and quantitation of nuclear and kinetoplast DNA was performed as previously described 26. We used 4′,6‐diamidino‐2‐phenylindole (DAPI) as the Adenine‐thymine (AT)‐selective stain and RedDot1 as the less sequence selective intercalating stain. To measure the relative binding of DAPI and RedDot1 to DNA with different sequence content, 21 random 100 bp‐long sequences were designed with AT content increasing at 5% intervals from 0 to 100%, and the forward and reverse sequences were synthesized as oligonucleotides. The lyophilized 100mer oligonucleotides were dissolved in annealing buffer (10 mM Tris·HCl pH 8.0, 50 mM NaCl and 1 mM EDTA) at 100 nM, mixed 1:1 with the appropriate reverse strand, and annealed into double stranded DNA by denaturation at 95 °C for 2 min followed by cooling to 25 °C over 45 min in a polymerase chain reaction machine. The double stranded DNA was diluted to 25 nM and mixed with 18 nM DAPI or 1× RedDot1 (supplied as a 200× stock, of unspecified concentration). Fluorescence with 358 nm excitation and 461 nm emission (DAPI) and 651 nm excitation and 694 nm emission (RedDot1) was measured using a SpectraMax M5 fluorescence plate reader (Molecular Devices). Background signal from an unstained sample was subtracted from the measurements.

Cells were washed by phosphate buffered saline (PBS) solution and centrifuged at 800*g* for 3 min; this step was repeated three times. Cells were then resuspended in 150 μL of PBS and the mixture was transferred to slides, which were left for 30 min at room temperature (RT) to allow cells to adhere. Due to the marine origin of *Perkinsela* and *D. papillatum*, 70% seawater was used instead of PBS in these steps. The remaining solution was washed off by PBS and slides were fixed in 4% paraformaldehyde for 30 min at RT. All paraformaldehyde was washed down by PBS and slides were immersed in −20 °C methanol and incubated for 4 h in a freezer. After this step, cells were rehydrated in PBS for 10 min. For RNA treatment, slides were incubated with RNAse A (50 μg/mL) for 2 h at RT and RNAse A was subsequently gently removed by incubation in PBS. Slides were stained by RedDot1 diluted 1:1,000 in PBS for 30 min according to the manufacturer's instructions. Fluorescent dye was washed and slides were mounted with mounting solution (Vectashield) containing DAPI. Representative areas in the mitochondrial or kinetoplast DNA and nuclear DNA of each species were selected manually as the reference values for color deconvolution. This organellar DNA was more AT‐rich than nuclear DNA in all species except *D. papillatum*. The AT content of the *C. helicis* kinetoplast DNA was only slightly more AT‐rich than the nuclear DNA, limiting the quality of color deconvolution. Relative quantity of kinetoplast DNA and nuclear DNA were measured from the integrated RedDot1 fluorescence intensity using manual area sections based on the color deconvolution result. For *Perkinsela* this measurement is likely an underestimate of the proportion of kinetoplast DNA; out‐of‐focus and scattered light from the immediately adjacent large kinetoplast DNA will have contributed to fluorescence signal at the position of the nucleus.

For transmission electron microscopy, cells were washed repeatedly in PBS, fixed in 2% glutaraldehyde in 0.2 M cacodylate buffer at 4 °C overnight, and processed as described previously 24. In the case of *Perkinsela*, high‐pressure freezing and freeze substitution were applied following the protocol described elsewhere 27.

## RESULTS AND DISCUSSION

### Quantification of Euglenozoan Mitochondrial Genomes

**Figure 1:**
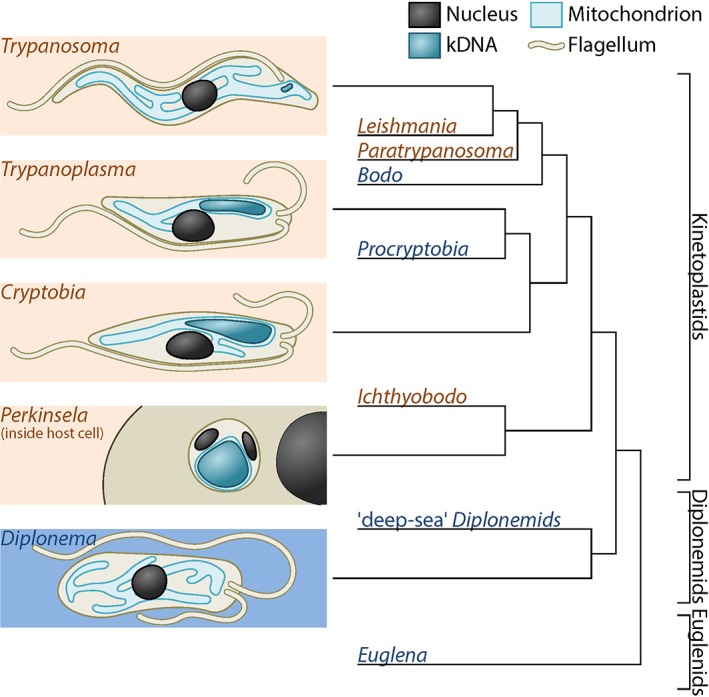
Diagram of the morphology and phylogeny of kinetoplastids, diplonemids, and euglenids. Cell morphology is shown on the left and a schematic phylogenetic tree is shown on the right. Brown genus names indicate parasitic and endosymbiotic species, blue indicate free‐living.

**Figure 2:**
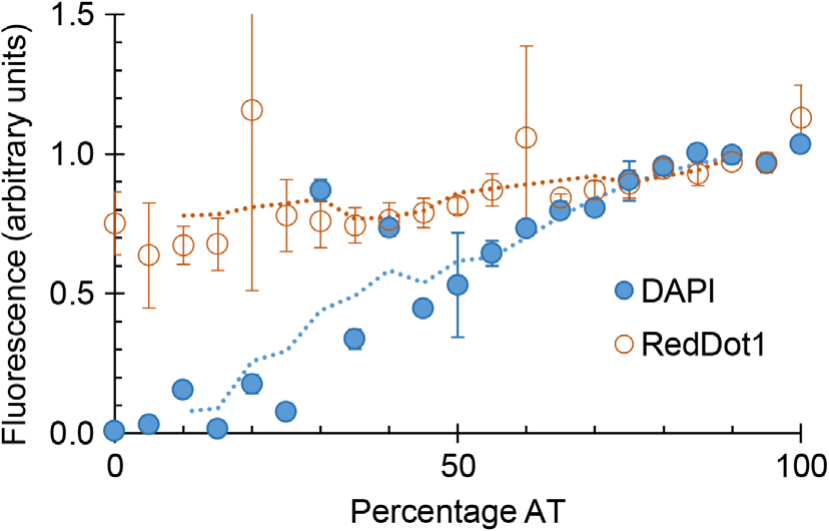
Relative binding of DAPI and RedDot1 stains to DNA. Fluorescence of 21 sequences 100 bp in length, with gradually increasing AT ratio and otherwise random sequence. The double stranded DNA was diluted to 25 nM, then mixed with DAPI and RedDot1 stains. Background‐corrected relative fluorescence is shown.

We determined the mitochondrial DNA content in the following representative kinetoplastids and diplonemids (Fig. 1): (i) the bovine blood parasite *Trypanosoma brucei* strain Lister 427 (cultured cells), (ii) the fish blood parasite *Trypanoplasma borreli* strain Tt‐JH (cultured cells), (iii) the snail parasite *Cryptobia helicis* (cells dissected from *Helix pomatia*), (iv) the amoeba endosymbiont *Perkinsela* sp. (*Paramoeba pemaquidensis* strain GillNOR1/1 was originally isolated from fish gill tissue), and (v) the aquatic plant pathogen *D. papillatum* strain ATCC50162 (cultured cells). Cells were subjected to differential staining with two chemically unrelated fluorescent dyes, DAPI and RedDot1. DAPI is a minor groove binding fluorescent stain, known to prefer AT‐rich sequences. RedDot1 is related to the Draq5 stain, being a less sequence‐sensitive base intercalator. We confirmed this by staining 100mer double stranded DNA with random sequences of AT content gradually increasing from 0 to 100% with DAPI and RedDot1 *in vitro*. DAPI signal fell to near zero at 0% AT. RedDot1 was relatively insensitive to sequence base composition, brightest at 100% AT and falling to 70% of that intensity at 0% AT (Fig. 2).

**Figure 3:**
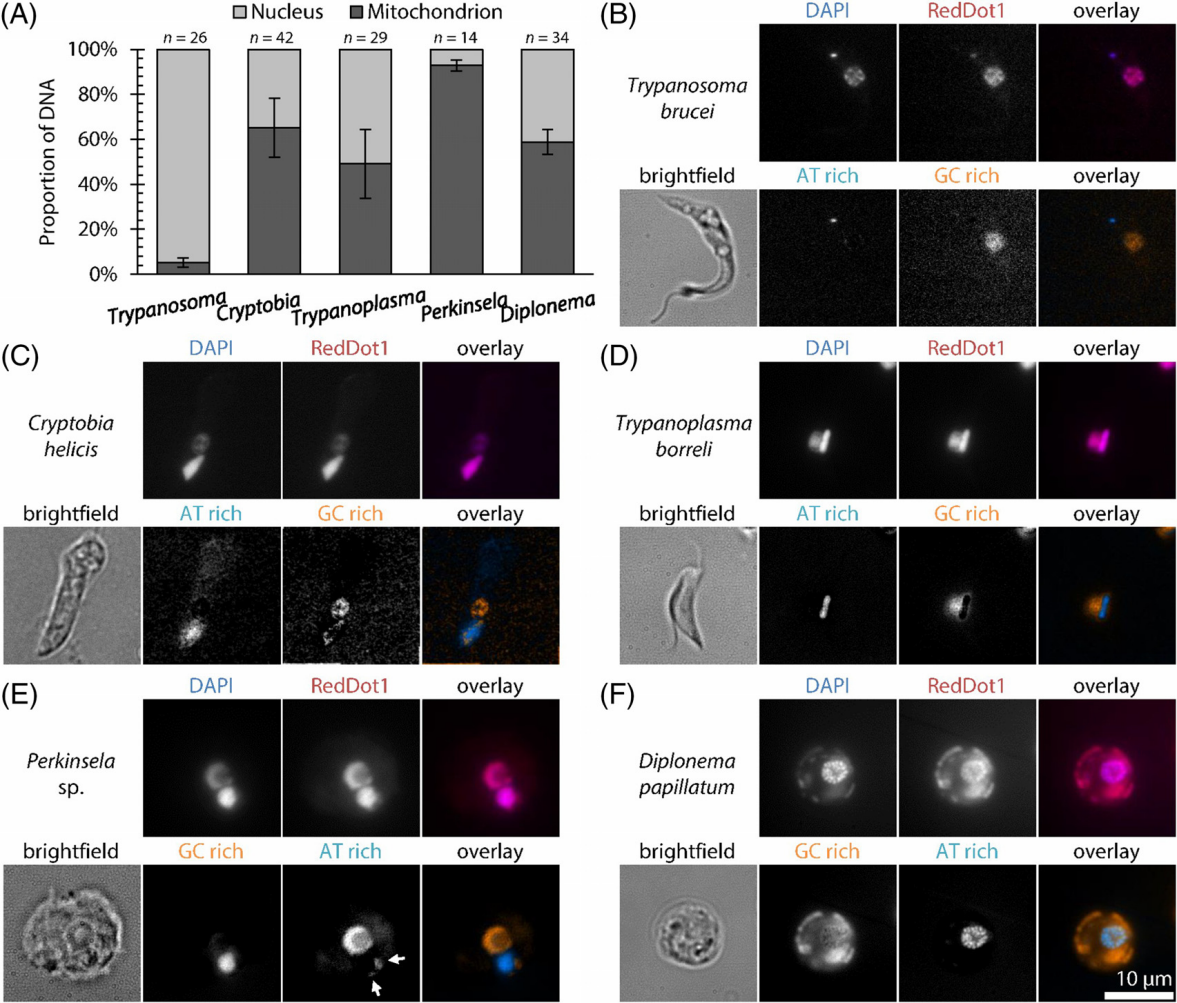
Ratio of nuclear to mitochondrial DNA in kinetoplastids and diplonemids. (A) Proportion of total cellular DNA found in the nucleus or mitochondrion was determined from integrated RedDot 1 fluorescence intensity in micrographs, in which color deconvolution of DAPI (AT‐selective) and RedDot 1 (less sequence‐specific) fluorescence signal was used to identify more AT‐rich or GC‐rich DNA and guide manual segmentation of the mitochondrial and nuclear DNA; see (B‑F). Error bars indicate standard error of proportion. An additional error of *ca*. ±15% from relative AT content of the mitochondrial and nuclear DNA is plausible. (B‑F) Example widefield epifluorescence micrographs of kinetoplastids and diplonemids stained with DAPI and RedDot, and the result of color deconvolution to identify more AT and more GC‐rich DNA in the cell. Kinetoplast DNA was more AT‐rich in all species except *D. papillatum* (F). *Perkinsela* sp. is an endosymbiont and both the host (large nucleus) and parasite nuclear DNA (arrows) are more GC‐rich (E).

The nuclear and mitochondrial (kinetoplast) DNA signals were distinguished by color deconvolution as previously demonstrated in *T. brucei* 26 (see Materials and Methods) (Fig. 3). The relative fluorescence of labeled genomes was quantified following manual segmentation of mitochondria and nuclei, and then measured as integrated RedDot1 signal intensity (Fig. 3A). In the case of *Perkinsela*, the signal corresponding to the host amoeba nucleus was excluded. The relative insensitivity of RedDot1 makes this DNA quantitation accurate, with a maximum error of *ca*. ±15% arising from the AT content of the DNA. In practice the maximum error is likely to be significantly smaller, for AT contents around 50%.

**Figure 4:**
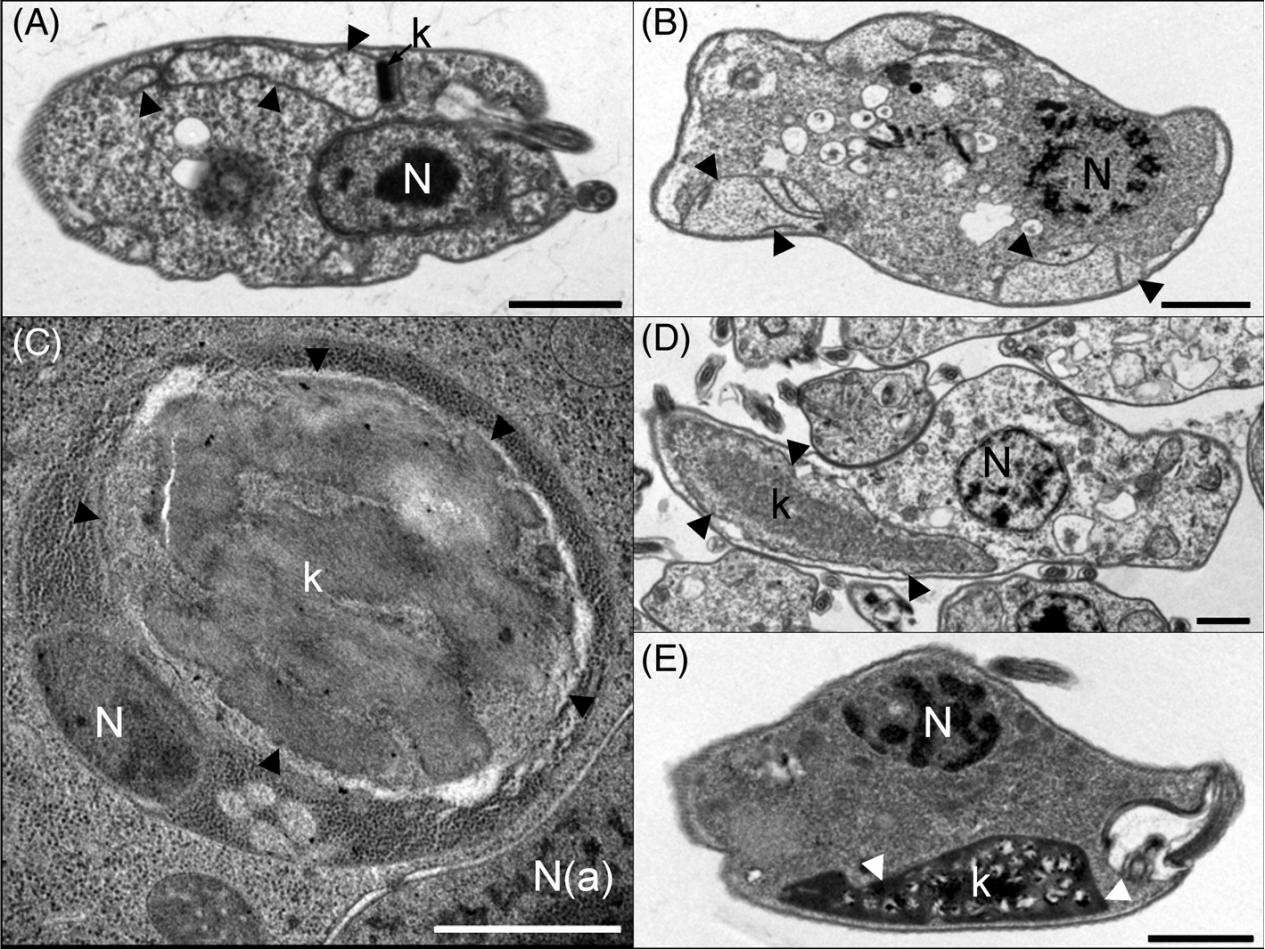
Relative size of the nuclear and mitochondrial DNA‐containing structures in kinetoplastids and diplonemids. Thin section transmission electron microscopy of (A) *Trypanosoma brucei*, (B) *Diplonema papillatum* (C) *Perkinsela* sp. (D) *Trypanoplasma borreli* and (E) *Cryptobia helicis*. The mitochondrion is indicated by arrowheads, the kDNA (present in all species except *D. papillatium*) is indicated by “k,” the nucleus is indicated by “N,” and the host cell nucleus (for *Perkinsela* sp.) is indicated by “N(a).” Scale bars represent 1 μm.

In line with DNA sequence data 21, 24, 28, the mitochondrial DNA of *D. papillatum* was more GC‐rich than its nuclear DNA (Fig. 3F). The kinetoplast DNAs of *T. brucei, T. borreli*, and *Perkinsela* were characteristically AT‐rich*,* with that of *C. helicis* being only moderately AT‐rich. Hence, due to the similar base composition of the nuclear and organellar DNAs, the color deconvolution protocol was slightly less effective for *C. helicis* (Fig. 3C). The more AT‐rich kinetoplast DNA within the large *Perkinsela* mitochondrion was located adjacent to one or two small patches of DNA of relatively lower AT content corresponding to the reduced *Perkinsela* nucleus (Fig. 3E). The amoeba host cell nucleus was also apparent. In agreement with electron microscopy (Fig. 4), the organellar DNA of *D. papillatum*, *T. borreli*, and *C. helicis* is dispersed through a large region of their reticulated mitochondria 14, while *T. brucei* has a canonical dot‐like periflagellar kinetoplast DNA 7.

### DNA Content and Ultrastructure

Our measurements indicated 5.09 ± 0.02% (mean ± standard error of proportion) of the total RedDot1 DNA stain fluorescent signal was in the dot‐like kinetoplast DNA of *T. brucei* (Fig. 3A; Table 1). The amount of kinetoplast DNA in this human pathogen is known only approximately, primarily based on the intensely studied kinetoplast DNA of *Crithidia fasciculata*, which is composed of ~5,000 minicircles and 40–50 maxicircles 7, 29. Extrapolating these numbers to *T. brucei* indicates 5,000 × 1.0 kilobase pairs (kbp) + 40 × 23.0 kbp, totaling approximately 5.9 megabase pairs (Mbp) of mitochondrial DNA. The diploid nuclear genome of *T. brucei* is estimated to be ~70 Mbp (http://www.sanger.ac.uk/resources/downloads/protozoa/trypanosoma-brucei.html) with an additional ~10% of nuclear DNA content made up of small‐sized and intermediate‐sized chromosomes of variable copy number 30. Thus the DNA content we measured (77.9 Mbp × 0.0509 / (1 − 0.0509) = 4.18 Mbp) is slightly less than expected, assuming the same kinetoplast DNA organization as *C. fasciculata,* and consistent with 0.0047 pg kinetoplast DNA and 0.097 pg nuclear DNA (0.001 pg ≈ 1 Mbp) previously measured by fluorimetry 31.

**Table 1:** DNA content and coding capacity of nuclear and mitochondrial genomes in kinetoplastid and diplonemid protists

	Nuclear DNA^a^		Mitochondrial (k) DNA	
Organism	Amount (Mbp)	Protein‐coding genes	Amount (Mbp)(SE)	Protein‐coding genes
*Trypanosoma brucei*	77.9	9,598	4.18 (±1.81)	18
*Cryptobia helicis*	n/d	n/d	~35.9	n/d
*Trypanoplasma borreli*	51.6	13,640	49.7 (±33.4)	~18
*Perkinsela* sp.	19.0	5,252	248.5 (±103.4)	6
*Diplonema papillatum*	~180.0	n/d	256.4 (±59.5)	18

a DNA amounts correspond to diploid genome size estimates inferred from sequence data. See text for references and further information (SE = standard errors).

The kinetoplast DNA of *C. helicis* was previously estimated to contain ~8,400 minicircles, each 4.2 kbp long, and 14 maxicircles each ~43 kbp in size 16. Combined, this amounts to ~36 Mbp. The proportion of total RedDot1 fluorescent signal in the kDNA in this species was found to be 58.7 ± 5.5% (Fig. 3C; Table 1). The size of its nuclear genome is unknown; inference of total DNA in the mitochondrion of *C. helicis* as above is thus not possible.

The kinetoplast DNA of *T. borreli*, which in DAPI‐stained cells appears as a spot significantly larger and somewhat brighter than the nucleus 14, contains ~200 kbp‐long molecules carrying standard mitochondrial‐encoded genes, the transcripts of which are extensively edited 25, 32. The diploid nuclear genome assembly of *T. borreli* is about 51.6 Mbp (33, Butenko et al., unpubl. data). The integrated intensity of RedDot1 fluorescence of the kDNA and nuclear DNA are approximately equal (kDNA proportion 49.1 ± 15.3%) (Fig. 3D; Table 1). Therefore, we estimate the total amount of *T. borreli* kDNA to be ∼50 Mbp.

The last examined kinetoplastid was the early branching *Perkinsela* sp. DAPI staining and electron microscopy revealed that this aflagellar endosymbiont contains significantly more DNA in its inflated mitochondrion than in its small nucleus 12, 17. The nuclear genome of *Perkinsela* was recently sequenced and found to be ~9.5 Mbp in size (diploid); with only 5,252 genes, it is the most reduced genome among this group of protists 27. Our quantitation showed the proportion of total cellular DNA in the kinetoplast was 92.8 ± 2.4% (Fig. 3E; Table 1). This indicates the DNA content in the organelle to be ∼120 Mbp, or ∼250 Mbp assuming both nuclei in the cell are diploid (see Materials and Methods). Electron microscopy is consistent with this estimation; circular cross‐sections through *Perkinsela* sp. kinetoplast DNA have a diameter of 2.01 ± 0.56 μm (*n* = 23), ~4 times larger than the corresponding DNA in *T. brucei*, indicating ~50–100 times more kinetoplast DNA in *Perkinsela* than *T. brucei* (assuming similar shape and packing density).

Finally, the well‐studied mitochondrial genome of *D. papillatum* contains >600 kbp of unique sequence, which must exist in many copies, as indicated by observed minor sequence polymorphisms 21. Based on the available assembly, the size of the diploid nuclear genome of *D. papillatum* is ~180 Mbp (Burger et al., unpubl. data). The presence of an extraordinarily large amount of DNA within its mitochondrion was clear from early cesium‐chloride density gradients combined with ethidium bromide and Hoechst 33258 dyes 34, and was further confirmed by DAPI‐staining and YOYO1‐staining of *D. papillatum* and other diplonemids, where a strong signal could be seen spread throughout the reticulated mitochondrion 24, 35. The proportion of total cellular DNA in the mitochondrion was 58.7 ± 5.5% (Fig. 3F; Table 1). We therefore estimate that the *D. papillatum* mitochondrion contains ∼260 Mbp of DNA.

Transmission electron microscopy revealed substantial differences in the fine structures of the mitochondrial DNAs examined herein. The well‐known kinetoplast DNA disk of *T. brucei* is electron‐dense due to the presence of taut, densely packed catenated minicircles (Fig. 4A), while the giant kDNA of *Perkinsela* appears to be comprised of large clumps of parallel DNA strands (Fig. 4C). Ultrastructurally, the kinetoplast DNA of *T. borreli* is visible as a uniformly dense cloud within the mitochondrial lumen (Fig. 4D), while the corresponding DNA of *C. helicis* also fills most of the mitochondrial matrix but has a different fine structure, forming numerous foci of varying electron density (Fig. 4E). In *D. papillatum*, dense structures likely corresponding to the mitochondrial DNA are visible among the large flat cristae (Fig. 4B). All things considered, the fine structures of these mitochondrial DNAs are very distinct from one other, which probably reflects differences in genome architecture and protein composition.

### Evolution of Mitochondrial Bizarreness in Kinetoplastids and Diplonemids

Mitochondrial genomes exist in a myriad of forms 36; they are well known for embellishing their structures and post‐transcriptional modifications 1. In terms of haploid genome size variation, however, mitochondrial genomes are relatively conservative. Here we have explored mitochondrial eccentricity from the perspective of DNA content per organelle.

In terms of organellar DNA size, the current record holder is the parasitic plant *Silene conica*, which has a mitochondrial genome of 11.3 Mbp, most of which has been acquired by horizontal gene transfer 41, 42. The largest plastid genomes sequenced thus far are the 1.13 Mbp genome of the red alga *Corynoplastis japonica* 37 and that of the green alga *Haematococcus lacustris*, whose genome is 1.35 Mbp in size 38. To our knowledge, total non‐nuclear DNA content has not been estimated for any of these organisms. In the case of *S. conica*, it is technically very challenging to separate the signal from the mitochondrion, plastid and nucleus using the experimental approaches used herein (our unpubl. data). At any rate, unless massively polyploid, the mitochondrion of *S. conica* contains less than the ~260 Mbp of DNA present in the mitochondrion of *D. papillatum*.

What might explain the ultra‐high quantity of DNA in the *D. papillatum* mitochondrion? An adaptive explanation is not obvious; in terms of gene content, it possesses a “typical” coding capacity of just 18 fragmented genes 20, 39. Even more extreme is the kinetoplast DNA of *Perkinsela*, which harbors 6.6 times more DNA than its corresponding nucleus, which may be related to the phenomenon of endosymbiont degradation 40. Electron microscopic studies from almost 50 years ago 41 revealed that some bodonid flagellates contain huge amounts of kinetoplast DNA. However, their size and coding capacity has only recently started to become clear 12, 27, 33. Only direct comparison with sequenced nuclear genomes, as carried out here, allows estimation of their size.

The textbook ratio of nuclear to non‐nuclear genomes in a “typical” eukaryote is >99% to <1% of total cellular DNA and protein‐coding genes 42. The quantity of DNA is essentially inverted in the endosymbiont *Perkinsela*, but the protein‐coding capacity is not: 5,252 and 6 genes are encoded by 9.5 and ~126.5 Mbp of DNA, respectively 12, 27. We do not know the copy number of the half‐dozen unique protein‐coding genes in its mitochondrial genome, but regardless, to the best of our knowledge such an imbalance is unprecedented.

Of what possible benefit could such large amounts of mitochondrial DNA be to kinetoplastids and diplonemids? Perhaps it serves as an organelle “filler” or as a novel form of energy storage (purine and pyrimidine catabolism could conceivably be used as an energy source). These adaptive explanations are purely speculative, and we default to the alternative view that such large amounts of organellar DNA are of no adaptive benefit at all. They may arise through truly neutral processes, or through constructive neutral evolution (CNE), which has been invoked to explain a variety of baroquely complex biological features. This includes mitochondrial transcript editing in kinetoplastids and gene scrambling in ciliates 43, 44, 45. CNE involves evolutionary ratchets that are likely to increase complexity through common mutations, while mutations which reverse this increased complexity are unlikely; CNE may have served to fuel the runaway expansion seen in kinetoplastid and diplonemid mitochondrial DNAs. However, although the DNA expansion itself might be fully explained by CNE, there is a debate as to whether this process is sufficient to explain the spread and distribution of pan‐editing in kinetoplastid parasites 46.

At present we can only speculate about the evolutionary forces driving the kinetoplastid and diplonemid protists on the path toward giant non‐nuclear DNA contents, especially given that their sister group, the euglenids, did not follow this path 18. Errors in DNA replication or segregation are unlikely to be responsible. A highly error‐prone mitochondrial DNA division mechanism, one with the potential to increase genome copy number to counter accidental loss of gRNA classes during division, is unlikely to be the cause, as random segregation of DNA molecules is a reliable mechanism for sufficiently high copy‐number molecules, based on molecule segregation during bacterial division 47. Moreover, the complexity of kinetoplast DNA division (involving >100 proteins in *T. brucei* 7) suggests non‐random segregation. An extremely high mitochondrial DNA mutation rate, that requires the existence of many copies of protein‐coding genes in order to maintain cell viability, also seems unlikely. The mutation rate necessary to disrupt all copies of a mitochondrial gene (likely present in thousands of copies given multi‐Mb mitochondrial DNA content) in a single generation would need to be phenomenally high, as high as in the most error‐prone viruses (Gago et al., 56).

This leaves several plausible evolutionary forces, such as hyperactive and/or expanded families of mitochondrial DNA polymerases leading to over‐duplication of mitochondrial DNA. Notably six mitochondrial DNA polymerases have been documented in *T. brucei* 48. Alternatively, massive RNA editing 9, 10 and/or *trans*‐splicing requiring huge coding space 21, 39 could act as an evolutionary ratchet for CNE 49. However, this could only be the case if extensive RNA editing existed before, or co‐evolved with, the runaway expansion of mitochondrial DNA (see below and Fig. 1).

In the case of *Perkinsela* sp., it is possible that its extreme kinetoplast DNA is somehow related to the fact that it is an endosymbiont, a lifestyle that is known to drive cells and their genomes in unusual directions 40, 50. In this regard, it will be important to explore the kinetoplast DNAs of *Ichthyobodo* spp., which are poorly studied ectoparasites of fish and the closest known relatives of *Perkinsela* spp. 51, 52. The nature of the kDNA in the common ancestor of these two lineages is an important missing piece of the puzzle. Regardless, as was shown recently, the kinetoplastid DNA of diplonemids is similarly extreme, and yet these organisms are among the most diverse and abundant eukaryotes in the world's oceans 53, 54. Diplonemids apparently have no large fitness cost of carrying the burden of a truly massive amount of non‐nuclear DNA. Clearly, we do not understand the energy costs of synthesizing so much seemingly useless DNA relative to the other aspects of their biology that impact evolutionary fitness. Diplonemids are thus a potentially useful system with which to study the evolutionary costs and benefits (if any) of giant extranuclear DNAs.

The most parsimonious interpretation of mitochondrial evolution in the lineages shown in Figure 1 is that organellar DNA content increased dramatically in a common ancestor shared by diplonemids and kinetoplastids soon after it diverged from *Euglena*. Thus, one can infer that *Trypanosoma* has either undergone massive reduction of its kinetoplast DNA or has not expanded it to the levels seen in its sister lineages. Why might this be so? Speijer suggested that parasitism could explain the rapid spread of pan‐editing in trypanosomatids and the “gene‐scrambling” it entails, and that intraspecific competition based on parasite replication rates could serve as an evolutionary counterforce leading to kinetoplast DNA loss 46, 55. What is needed to test these hypotheses is more fine‐scale information on the relationship between extranuclear DNA content and RNA editing levels. Do organisms that encounter strong intraspecific competition in hosts indeed have less mitochondrial DNA and the highest levels of pan‐editing? If so, CNE may be only part of the explanation for the evolution of these exceedingly complex, almost burlesque genetic systems. We are in urgent need of more data from the free‐living diplonemids and parasites such as *Ichthyobodo*.
